# Texting Condolences: Adapting mHealth Programs After Unexpected Pregnancy and Infant Outcomes

**DOI:** 10.2196/mhealth.8303

**Published:** 2017-12-08

**Authors:** Jennifer A Unger, John Kinuthia, Grace John-Stewart

**Affiliations:** ^1^ Global Center for Integrated Health of Women, Adolescents and Children Department of Global Health University of Washington Seattle, WA United States; ^2^ Department of Obstetrics and Gynecology University of Washington Seattle, WA United States; ^3^ Department of Research and Programs Kenyatta National Hospital Nairobi Kenya; ^4^ Department of Obstetrics and Gynaecology Kenyatta National Hospital Nairobi Kenya

**Keywords:** mHealth, infant loss, miscarriage

## Abstract

Mobile health (mHealth) short message system (SMS) interventions for maternal and child health (MCH) are being implemented globally. In many low- and middle-income settings in which these mHealth interventions are being rolled out, stillbirths and neonatal and infant deaths are common. It is important that mHealth solutions do not exacerbate emotional stress and pain by continuing with routine messaging for pregnancy or infant care when someone has experienced loss. In this brief viewpoint paper, we argue that SMS programs for maternal and child health need to adapt and make available messaging for miscarriage, stillbirth, and infant loss.

Mobile health (mHealth) short message system (SMS) text messaging interventions such as Text4baby [1] and MomConnect [2] are increasingly incorporated within maternal child health (MCH) care systems globally. Evidence regarding effectiveness of these interventions to improve MCH health services uptake and outcomes is limited to date but accruing [3-5]. Studies suggest that these interventions may improve beliefs and attitudes about behaviors and preparedness during pregnancy [1,6] and that women appreciate access to information [7] throughout pregnancy and the postpartum period. Message development for MCH text message programs has involved tailoring messaging to the stage of pregnancy and infant age and targeting key behaviors that can improve health outcomes. As women connect to the health care system through text messaging, there is increasing programmatic responsibility to get the messaging right for these participants. Messaging needs to be culturally appropriate, accurate, and reflect realities to be a credible resource that has positive impact on maternal and child outcomes.

An unanticipated challenge we faced during a recent mHealth randomized controlled trial (RCT), Mobile WACh (Mobile Solutions for Women’s and Children’s Health) [NCT01894126], was responding to the high number of neonatal and infant deaths, stillbirths, or miscarriages. Unfortunately, in many low- and middle-income settings in which mHealth interventions are being rolled out, stillbirths and neonatal and infant deaths are not infrequent [8]. Although mHealth MCH systems automate text messaging based on timing of gestation or postpartum status, most are not built to capture the real-time vital status of mother or infant. Thus, a woman may continue to receive messages from the program about actions to keep her pregnancy or baby healthy after the occurrence of a miscarriage, stillbirth, or infant death. Women could end messaging by sending a STOP message, but this leaves these women without access to a potentially valuable source of support. Women with adverse outcomes may especially benefit from access to advice and encouragement at this difficult time. In our RCT, we found that when we contacted women for study follow-up, most women who experienced a stillbirth or infant death wanted to continue receiving messages. In addition, women in the bidirectional text messaging group wanted to be able to continue a message dialog with the nurse.

**Example text messages for women participating in mHealth programs after a miscarriage or infant death.**

Immediate message after infant loss:

[Name], this is [nurse] from [clinic]. We are very sorry to hear about your loss. It may be more difficult to take care of yourself but you need to make sure to take time each day for your health, even now. If you have any questions or concerns, please come in to the clinic. We are all here for you.

Week 1 postloss:

[Name], this is [nurse] from [clinic]. It may be difficult to cope after your loss. Try and do something each day to take care of yourself. Talk to your family and friends or go for a walk. Please come in and talk to the nurses if it helps.

Week 3 postloss:

[Name], this is [nurse] from [clinic]. It may be a very difficult time after your loss. Please continue to take care of yourself and know we are thinking of you. Please come in to the clinic if we can be of help.

Week 4 postloss:

[Name], this is [nurse] from [clinic]. It may be best to delay your next pregnancy just to make sure you are healthy and feeling your best. Even if you don’t want to go to the maternal child health clinic, we can send you information about family planning or you can get family planning from another clinic.

With scale-up of mHealth in MCH systems, the intersection between messaging projects and infant deaths is not insignificant. For example, South Africa has rolled out MomConnect countrywide to improve maternal and child outcomes. The infant mortality rate in South Africa is 33 infant deaths per 1000 live births [9], and MomConnect has messaged over 700,000 pregnant women since its launch in 2013. Thus, an estimated 23,100 women receiving MCH-related text messages will also experience the loss of their baby. It is important that mHealth solutions do not exacerbate emotional stress and pain with perhaps unwanted reminders of this loss, and programs need to be thoughtful about how these women and their families could benefit from adapted messages tailored to this scenario.

One approach that may also lessen the stigma of miscarriage, stillbirth, and infant death is to develop messaging around these sad events (Textbox 1). The messages in our trial were developed and revised after formative work, both pre-RCT focus group discussions and post-RCT interviews, with representative women from the communities where we work. Although there are limitations to all counseling messages and they may not address the concerns of all women, messaging could be designed to support women and encourage healthy behaviors around grief and depression. Women would then have the option to remain connected to the program and not experience a loss of support. Building such a system would require some design innovations to enable prompt notification of infant deaths by integrating more closely with primary health care workers but could ultimately provide valuable information about infant deaths and the needs and experiences of participating women and result in long-term improved outcomes for these families.

## Abbreviations

MCH: maternal child health
Mobile WACh: Mobile Solutions for Women’s and Children’s Health
RCT: randomized controlled trial
SMS: short message service
